# Comparison of SGLT2 inhibitors vs. DPP4 inhibitors for patients with metabolic dysfunction associated fatty liver disease and diabetes mellitus

**DOI:** 10.1007/s40618-023-02246-6

**Published:** 2023-12-19

**Authors:** Y. Suzuki, H. Kaneko, A. Okada, R. Ohno, I. Yokota, K. Fujiu, T. Jo, N. Takeda, H. Morita, K. Node, H. Yasunaga, I. Komuro

**Affiliations:** 1 The Department of Cardiovascular Medicine, The University of Tokyo, 7-3-1, Hongo, Bunkyo-ku, Tokyo, 113-8655 Japan; 2 Center for Outcomes Research and Economic Evaluation for Health, National Institute of Public Health, Saitama, Japan; 3 The Department of Advanced Cardiology, The University of Tokyo, Tokyo, Japan; 4 Department of Prevention of Diabetes and Lifestyle-Related Diseases, Graduate School of Medicine, The University of Tokyo, Tokyo, Japan; 5 Department of Biostatistics, Faculty of Medicine, Hokkaido University, Sapporo, Japan; 6 The Department of Health Services Research, The University of Tokyo, Tokyo, Japan; 7 Department of Cardiovascular Medicine, Saga University, Saga, Japan; 8 Department of Clinical Epidemiology and Health Economics, School of Public Health, The University of Tokyo, Tokyo, Japan; 9 International University of Health and Welfare, Tokyo, Japan; 10 Department of Frontier Cardiovascular Science, Graduate School of Medicine, The University of Tokyo, Tokyo, Japan

**Keywords:** SGLT2 inhibitors, Fatty liver disease, MAFLD, Diabetes mellitus, Liver enzymes

## Abstract

**Purpose:**

This study aimed to examine the potential benefit of sodium-glucose cotransporter 2 (SGLT2) inhibitors for patients with metabolic dysfunction-associated fatty liver disease (MAFLD) and diabetes mellitus (DM) using a real-world database.

**Methods:**

We analyzed individuals with MAFLD and DM newly initiated on SGLT2 or dipeptidyl peptidase 4 (DPP4) inhibitors from a large-scale administrative claims database. The primary outcome was the change in the fatty liver index (FLI) assessed using a linear mixed-effects model from the initiation of SGLT2 or DPP4 inhibitors. A propensity score-matching algorithm was used to compare the change in FLI among SGLT2 and DPP4 inhibitors.

**Results:**

After propensity score matching, 6547 well-balanced pairs of SGLT2 and 6547 DPP4 inhibitor users were created. SGLT2 inhibitor use was associated with a greater decline in FLI than DPP4 inhibitor use (difference at 1-year measurement, − 3.8 [95% CI − 4.7 to − 3.0]). The advantage of SGLT2 inhibitor use over DPP4 inhibitor use for improvement in FLI was consistent across subgroups. The relationship between SGLT2 inhibitors and amelioration of FLI was comparable between individual SGLT2 inhibitors.

**Conclusions:**

Our analysis using large-scale real-world data demonstrated the potential advantage of SGLT2 inhibitors over DPP4 inhibitors in patients with MAFLD and DM.

**Supplementary Information:**

The online version contains supplementary material available at 10.1007/s40618-023-02246-6.

## Introduction

The clinical implications of a novel concept named “metabolic dysfunction-associated fatty liver disease” (MAFLD) intrigue clinical interest. FLD is becoming more common and a serious public health concern. A global panel of experts proposed the new terminology MAFLD in 2020 [1], which replaced the term “non-alcoholic fatty liver disease” (NAFLD). Regardless of alcohol consumption habits, MAFLD has its own inclusion criteria based on a number of metabolic abnormalities. Diabetes mellitus (DM) is a metabolic abnormality included in the diagnostic criteria for MAFLD. Reduction in body weight can bring about improvements in glucose homeostasis and lessen cardiometabolic risk factors in patients with DM; however, lifestyle‐based weight loss interventions (e.g., exercise, diet, and behavior modification) may not be effective in the long term [2]. On the other hand, a recent systematic review shows that some glucose-lowering drugs are effective in inducing weight loss in patients with DM [3]. Sodium-glucose cotransporter 2 (SGLT2) inhibitors (inhibiting the reabsorption of glucose in the proximal tubule, resulting in the promotion of urinary glucose excretion and improvement in glycemic control) were originally developed as drugs for DM. Recent clinical trials have demonstrated the robust cardiovascular and kidney protective effects of SGLT2 inhibitors [4–14]. Furthermore, several small clinical trials have shown that SGLT2 inhibitors could also be effective against FLD [15–18]. However, little is known regarding whether the results of previous RCTs focusing on the advantage of SGLT2 inhibitors for FLD could be applicable to a broader range of patients with DM and MAFLD encountered in real-world clinical practice. Therefore, we analyzed a nationwide epidemiological database and sought to validate the potential benefits of SGLT2 inhibitor use in patients with DM and MAFLD.

## Materials and methods

Anonymized data are publicly available for purchase from JMDC Inc.

### Study population

This retrospective cohort study used the JMDC Claims Database, a large-scale administrative claims database [19–21]. The JMDC includes annual health checkup data (e.g., blood tests and anthropometric measurements) and health insurance records between 2005 and 2022. In Japan, annual health checkups for employees are a legal requirement. The JMDC Claims Database accumulates insurance claims data. Medical diagnoses were coded according to the International Classification of Diseases, 10th revision (ICD-10). We extracted the data of 21,883 individuals with DM (ICD-10 codes E10–E14) and MAFLD defined as ICD-10 codes of E10–E14 and fatty liver index (FLI) ≥ 30 [22, 23], who newly initiated SGLT2 (empagliflozin, dapagliflozin, canagliflozin, ipragliflozin, tofogliflozin, and luseogliflozin) or dipeptidyl peptidase 4 (DPP4) inhibitors (Supplementary Fig. 1). FLI was calculated using the following formula: FLI = 1/1 + e^−(0.953×ln(triglycerides) + 0.139×(BMI) + 0.718×ln(γ−glutamyl transpeptidase [γ−GTP]) + 0.053×(waist circumference)−15.745)^ × 100. We defined initiating either drug class among individuals who had not previously used either drug class within the previous year as a new use. Furthermore, only individuals with available repeated data for the assessment of FLI during health checkups were included in this study. Among 21,883 individuals, we excluded participants for the following reasons: age < 20 years (n = 1); a history of liver disease defined as liver cancer (ICD-10 code: C22), fibrosis and cirrhosis of the liver (ICD-10 code: K74), hepatitis B (ICD-10 code: B16), hepatitis C (ICD-10 code: B182), autoimmune hepatitis (ICD-10 code: K754), and cholangitis (ICD-10 code: K830) (n = 317); and missing cigarette smoking (n = 262), alcohol consumption (n = 1257), and physical activity (n = 521) data. Finally, 19,525 individuals were included in this study (Supplementary Fig. 2).

### Ethics

This study was approved by the Ethics Committee of the University of Tokyo (approval number: 2018-10862), and informed consent was not required because all data included in the JMDC Claims Database were anonymized and de-identified.

### Measurements and definitions

We obtained the following data from the health checkups: body mass index (BMI), waist circumference, blood pressure, laboratory data (fasting plasma glucose, hemoglobin A1c [HbA1c], low-density lipoprotein cholesterol, high-density lipoprotein cholesterol, triglycerides, aspartate aminotransferase [AST], alanine aminotransferase [ALT], and gamma-glutamyl transpeptidase [γ-GTP]), cigarette smoking (current or noncurrent/never), alcohol consumption (daily or not every day), and physical activity (active or inactive). Cigarette smoking and alcohol consumption were assessed using a self-report questionnaire during the health checkup. Physical inactivity was defined as not exercising for 30 min ≥ 2 times a week or not walking for more than an hour per day. Based on the ICD-10 code, we obtained data on the presence of diabetic nephropathy (ICD-10 codes: E102, E112, E122, E132, and E142), diabetic retinopathy (ICD-10 codes E103, E113, E123, E133, and E143), and diabetic neuropathy (ICD-10 codes: E104, E114, E124, E134, and E144) at the prescription date of SGLT2 or DPP4 inhibitors. Data on concomitant medications at the prescription date of SGLT2 or DPP4 inhibitors were extracted from administrative claims records.

### Propensity score matching

A propensity score matching algorithm was used to generate a matched cohort to compare the benefits of SGLT2 and DPP4 inhibitor use. We estimated the propensity score for SGLT2 inhibitor users using a logistic regression model. To estimate the propensity score, we included the following variables: age, sex, BMI, waist circumference, systolic blood pressure, diastolic blood pressure, fasting plasma glucose, HbA1c, low-density lipoprotein cholesterol, high-density lipoprotein cholesterol, triglycerides, AST, ALT, γ-GTP, cigarette smoking, alcohol consumption, physical inactivity, diabetic nephropathy, diabetic retinopathy, diabetic neuropathy, use of medications (insulin, glucagon-like peptide-1 receptor agonist, biguanide, sulfonylurea, α-glucosidase inhibitor, thiazolidine, glinide, renin-angiotensin system inhibitor, β-blocker, calcium channel blocker, mineralocorticoid receptor antagonist, diuretics, and statins), year of SGLT2 or DPP4 inhibitors prescription, and FLI at the initial health checkup. We matched SGLT2 and DPP4 inhibitor users using a 1:1 matching protocol (caliper width equal to 0.2 standard deviations of the logit score).

### Outcomes

Outcomes were obtained from the annual health checkup data between 2005 and 2022. The primary outcome was the change in FLI after the initiation of SGLT2 or DPP4 inhibitors. The secondary outcomes were changes in γ-GTP, AST, ALT, BMI, waist circumference, and HbA1c levels. We followed the study participants for a maximum of 5 years after the initial health checkup.

### Statistical analysis

The median (interquartile range) and number (percentage) were used to report descriptive statistics. We used a linear mixed-effects model for repeated measures with random intercept and slope, assuming an unstructured covariance structure, to compare the change in outcomes, including FLI, γ-GTP, AST, ALT, BMI, waist circumference, and HbA1c, among SGLT2 and DPP4 inhibitors. This model included the treatment group (SGLT2 or DPP4 inhibitors), time, and interaction between the treatment group and time. To examine the difference in the outcome changes among SGLT2 or DPP4 inhibitors, the *P*-value for the interaction between the treatment group and time was tested using Wald test.

If a significant difference in the primary outcome change was detected between SGLT2 and DPP4 inhibitors, we compared the change in FLI between individual SGLT2 inhibitors to examine whether the effects of SGLT2 inhibitors would be considered a class effect. We also performed a linear mixed-effects model for repeated measures to compare the change in FLI among empagliflozin, dapagliflozin, canagliflozin, ipragliflozin, tofogliflozin, and luseogliflozin. Presently, these six SGLT2 inhibitors are commercially available in Japan.

This model included individual SGLT2 inhibitors, time, the interaction between the individual SGLT2 inhibitors and time, age, sex, BMI, waist circumference, systolic blood pressure, diastolic blood pressure, fasting plasma glucose, HbA1c, low-density lipoprotein cholesterol, high-density lipoprotein cholesterol, triglycerides, AST, ALT, γ-GTP, cigarette smoking, alcohol consumption, physical inactivity, diabetic nephropathy, diabetic retinopathy, diabetic neuropathy, use of the following medications (insulin, glucagon-like peptide-1 receptor agonist, biguanide, sulfonylurea, α-glucosidase inhibitor, thiazolidine, glinide, renin-angiotensin system inhibitor, β-blocker, calcium channel blocker, mineralocorticoid receptor antagonist, diuretics, and statins), and year of SGLT2 inhibitor prescription. To examine the difference in outcome changes among individual SGLT2 inhibitors, the *P-*value for the interaction between individual SGLT2 inhibitors and time was tested using a Wald test.

Three sensitivity analyses were performed to validate the primary findings. First, we examined the changes in FLI only in individuals who continued to use SGLT2 or DPP4 inhibitors for > 3 months. Second, we performed a linear mixed-effects model using a restricted cubic spline function with 4 knots to confirm the shape of the change in FLI from the initiation of SGLT2 inhibitors or DPP4 inhibitors. Third, we performed subgroup analyses stratified by age (≥ 50 and < 50 years), sex, BMI (≥ 30 and < 30 kg/m^2^), and HbA1c level at the initial health checkup (≥ 7.5 and < 7.5%). All statistical analyses were performed using STATA version 17 (StataCorp LLC, College Station, TX, USA).

## Results

### Clinical characteristics

Table 1 shows the baseline clinical characteristics of study participants before and after propensity score matching. After 1:1 propensity score matching, 6547 pairs were created. After propensity score matching, the individual distributions were well balanced between SGLT2 and DPP4 inhibitor users. The median age was 51 (46–56) years for SGLT2 inhibitor users and 51 (45–56) years for DPP4 inhibitor users. In addition, 5476 (83.6%) individuals were men in SGLT2 inhibitors users, and 5449 (83.2%) individuals were men in DPP4 inhibitors users. The median FLI was 78.2 (59.1–90.9) in SGLT2 inhibitor users and 78.7 (59.5–91.2) in DPP4 inhibitor users.

**Table 1:** Baseline Characteristics

	Before propensity score matching			After propensity score matching		
	DPP4 inhibitors (n = 12,210)	SGLT2 inhibitors (n = 7315)	SMD	DPP4 inhibitors (n = 6547)	SGLT2 inhibitors (n = 6547)	SMD
Age, years	52 (47–58)	51 (45–56)	− 0.223	51 (45–56)	51 (46–56)	0.006
Men, n (%)	10,333 (84.6)	6078 (83.1)	− 0.042	5449 (83.2)	5476 (83.6)	0.011
BMI, kg/m^2^	27.5 (25.2–30.5)	29.4 (26.7–32.8)	0.420	29.0 (26.4–32.3)	29.0 (26.5–32.1)	− 0.024
Waist Circumference, cm	94.0 (88.2–101.0)	98.0 (91.5–106.0)	0.392	97.0 (90.8–105.0)	97.3 (91.0–104.6)	− 0.022
SBP, mmHg	131 (122–142)	130 (121–141)	− 0.036	130 (122–141)	130 (121–142)	− 0.002
DBP, mmHg	83 (76–91)	83 (76–90)	− 0.015	83 (76–91)	83 (76–91)	− 0.003
Cigarette Smoking, n (%)	4450 (36.4)	2350 (32.1)	− 0.091	2148 (32.8)	2188 (33.4)	0.013
Alcohol Consumption, n (%)	3026 (24.8)	1432 (19.6)	− 0.126	1345 (20.5)	1360 (20.8)	0.006
Physical inactivity, n (%)	7302 (59.8)	4449 (60.8)	0.021	3970 (60.6)	3960 (60.5)	− 0.003
Comorbidity						
Diabetic nephropathy, n (%)	665 (5.4)	682 (9.3)	0.149	485 (7.4)	475 (7.3)	− 0.006
Diabetic retinopathy, n (%)	991 (8.1)	937 (12.8)	0.154	714 (10.9)	695 (10.6)	− 0.009
Diabetic neuropathy, n (%)	143 (1.2)	182 (2.5)	0.098	120 (1.8)	119 (1.8)	− 0.001
Medication						
Insulins, n (%)	409 (3.3)	530 (7.2)	0.175	335 (5.1)	340 (5.2)	0.003
GLP-1 Receptor Agonist, n (%)	54 (0.4)	228 (3.1)	0.203	54 (0.8)	54 (0.8)	0.000
Biguanide, n (%)	2135 (17.5)	1827 (25.0)	0.184	1475 (22.5)	1469 (22.4)	− 0.002
Sulfonylurea, n (%)	492 (4.0)	300 (4.1)	0.004	257 (3.9)	247 (3.8)	− 0.008
α-GI, n (%)	379 (3.1)	253 (3.5)	0.020	215 (3.3)	205 (3.1)	− 0.009
Thiazolidine, n (%)	200 (1.6)	238 (3.3)	0.105	162 (2.5)	163 (2.5)	0.001
Glinides, n (%)	91 (0.7)	78 (1.1)	0.034	60 (0.9)	54 (0.8)	− 0.010
Renin angiotensin system inhibitor, n (%)	3764 (30.8)	2769 (37.9)	0.148	2347 (35.8)	2343 (35.8)	− 0.001
Beta-blocker, n (%)	778 (6.4)	668 (9.1)	0.103	532 (8.1)	527 (8.0)	− 0.003
Calcium channel blocker, n (%)	2870 (23.5)	1848 (25.3)	0.041	1609 (24.6)	1614 (24.7)	0.002
Mineralocorticoid receptor antagonist, n (%)	159 (1.3)	180 (2.5)	0.085	130 (2.0)	129 (2.0)	− 0.001
Diuretics, n (%)	782 (6.4)	673 (9.2)	0.104	554 (8.5)	537 (8.2)	− 0.009
Statin, n (%)	3162 (25.9)	2266 (31.0)	0.113	1934 (29.5)	1932 (29.5)	− 0.001
Laboratory Data						
Glucose, mg/dL	142 (124–175)	136 (118–166)	− 0.137	139 (122–166)	137 (118–167)	− 0.003
HbA1c, %	7.3 (6.7–8.4)	7.1 (6.5–8.2)	− 0.143	7.2 (6.7–8.1)	7.1 (6.5–8.2)	− 0.007
LDL-C, mg/dL	132 (110–156)	129 (107–153)	− 0.078	130 (108–153)	130 (108–154)	0.006
HDL-C, mg/dL	49 (42–57)	48 (42–56)	− 0.054	48 (42–56)	48 (42–56)	0.000
Triglycerides, mg/dL	161 (116–233)	158 (112–226)	− 0.034	160 (115–230)	158 (113–228)	0.005
AST, U/L	28 (21–41)	31 (22–45)	0.137	30 (22–45)	30 (22–44)	− 0.008
ALT, U/L	39 (26–62)	44 (28–71)	0.174	44 (28–71)	44 (28–70)	− 0.006
γ-GTP, U/L	58 (39–95)	58 (38–92)	− 0.042	59 (39–95)	59 (38–93)	− 0.010
FLI	71.3 (52.1–87.1)	80.0 (60.7–92.2)	0.309	78.7 (59.5–91.2)	78.2 (59.1–90.9)	− 0.026

Data are reported as medians (interquartile range) or numbers (percentage), where appropriate

*DPP-4* dipeptidyl peptidase-4, *SGLT2* sodium-glucose cotransporter-2, *BMI* body mass index, *SBP* systolic blood pressure, *DBP* diastolic blood pressure, *GLP-1* glucagon-like peptide 1, *α-GI* α-glucosidase inhibitor, *LDL-C* low-density lipoprotein cholesterol, *HDL-C* high-density lipoprotein cholesterol, *AST* aspartate aminotransferase, *ALT* alanine aminotransferase, *γ-GTP* γ-glutamyl transpeptidase, *FLI* fatty liver index

### Change in outcomes among SGLT2 and DPP4 inhibitors

**Fig. 1:**
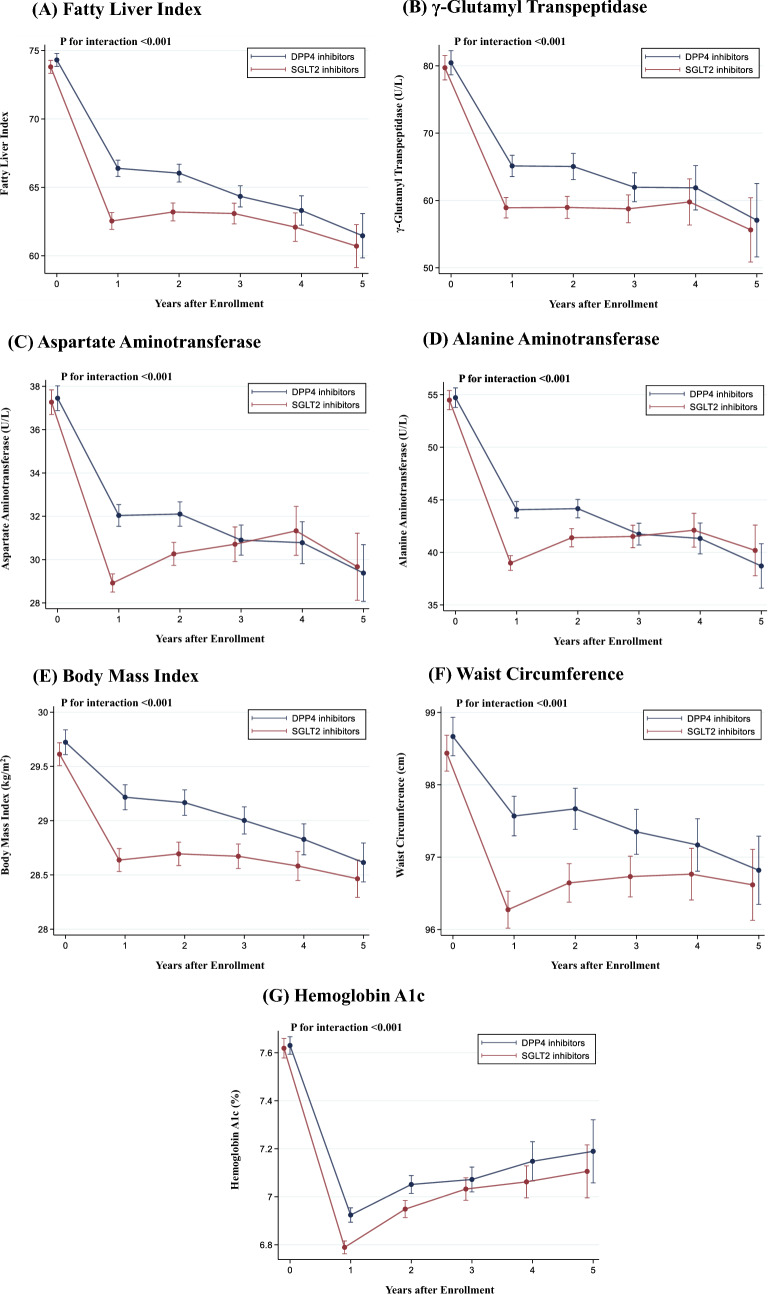
Comparison of the Change in Outcomes among SGLT2 Inhibitors and DPP4 Inhibitors. We performed a linear mixed-effects model to compare the change in fatty liver index (**A**), γ-glutamyl transpeptidase (**B**), aspartate aminotransferase (**C**), alanine aminotransferase (**D**), body mass index (**E**), waist circumference (**F**), and hemoglobin A1c (**G**) among SGLT2 inhibitors and DPP4 inhibitors. The model included treatment group (SGLT2 inhibitors or DPP4 inhibitors), time, and the interaction between the treatment group and time. Error bars represented a 95% confidence interval

The mean follow-up period was 750 ± 437 days. Figure 1 shows the changes in outcomes after the initiation of SGLT2 or DPP4 inhibitors. SGLT2 inhibitor users showed a greater decline in FLI than that of DPP4 inhibitor users. The predicted difference in FLI among SGLT2 and DPP4 inhibitors at 1-year measurement was -3.8 (95% confidence interval [95% CI], − 4.7 to − 3.0). A significant interaction was detected between the treatment group (SGLT2 or DPP4 inhibitors) and the time spent on FLI (*P* for interaction < 0.001).

Furthermore, SGLT2 inhibitors users had a greater decline in γ-GTP, AST, ALT, BMI, waist circumference, and HbA1c at 1-year measurement than that of DPP4 inhibitors users (predicted difference [95% CI]; − 6.2 [95% CI − 8.4 to − 4.0] U/L; − 3.1 [95% CI − 3.8 to − 2.5] U/L; − 5.1 [95% CI − 6.1 to − 4.0] U/L; − 0.6 [95% CI − 0.7 to − 0.4] kg/m^2^; − 1.3 [95% CI − 1.7 to − 0.9] cm; and − 0.1% [95% CI − 0.2 to − 0.1], respectively). We detected a significant interaction between the treatment group (SGLT2 or DPP4 inhibitors) and time on γ-GTP, AST, ALT, BMI, waist circumference, and HbA1c (all *P* < 0.001).

### Change in FLI among individual SGLT2 inhibitors

**Fig. 2:**
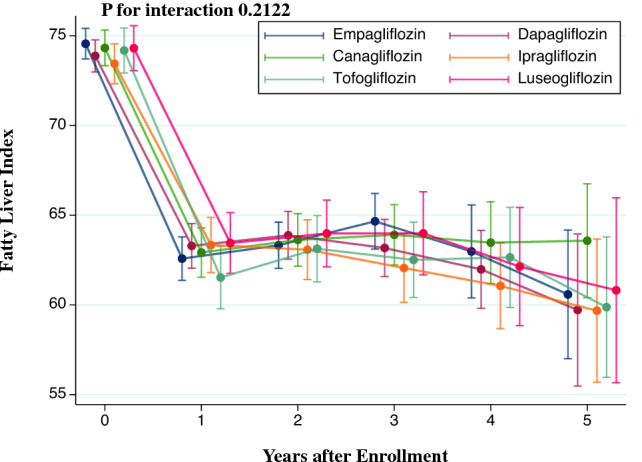
Comparison of the Change in Fatty Liver Index among SGLT2 Inhibitors. We performed a linear mixed-effects model to compare the change in fatty liver index among individual SGLT2 inhibitors. The model included individual SGLT2 inhibitors, time, the interaction between the individual SGLT2 inhibitors and time, age, sex, systolic blood pressure, diastolic blood pressure, fasting plasma glucose, low-density lipoprotein cholesterol, high-density lipoprotein cholesterol, triglycerides, cigarette smoking, alcohol consumption, physical inactivity, diabetic nephropathy, diabetic retinopathy, diabetic neuropathy, use of the following medications (insulin, glucagon-like peptide-1 receptor agonist, biguanide, sulfonylurea, α-glucosidase inhibitor, thiazolidine, glinide, renin-angiotensin system inhibitor, β-blocker, calcium channel blocker, mineralocorticoid receptor antagonist, diuretics, and statin), year at the prescription of SGLT2 inhibitors. Error bars represented a 95% confidence interval

We compared the change in FLI between individual SGLT2 inhibitors because of the significant difference in FLI change among SGLT2 and DPP4 inhibitors. We analyzed 6,535 SGLT2 inhibitor users after excluding individuals prescribed multiple SGLT2 inhibitors (n = 12) from 6547 SGLT2 inhibitor users. SGLT2 inhibitor users were categorized into six groups: empagliflozin (n = 1593), dapagliflozin (n = 1389), canagliflozin (n = 1137), ipragliflozin (n = 955), tofogliflozin (n = 703), and luseogliflozin (n = 758). Figure 2 shows the change in FLI after the initiation of SGLT2 inhibitors. Each SGLT2 inhibitor user showed a similar reduction in FLI. The differences in FLI at 1-year measurement were 0.7 (95% CI − 1.0 to 2.4) for dapagliflozin, 0.3 (95% CI − 1.5 to 2.2) for canagliflozin, 0.8 (95% CI − 1.2 to 2.7) for ipragliflozin, − 1.1 (95% CI − 3.2 to 1.1) for tofogliflozin, and 0.9 (95% CI − 1.2 to 2.9) for luseogliflozin compared with empagliflozin. We detected no statistically significant interaction between individual SGLT2 inhibitors and time on the FLI (*P* = 0.2122).

### Sensitivity analyses

**Fig. 3:**
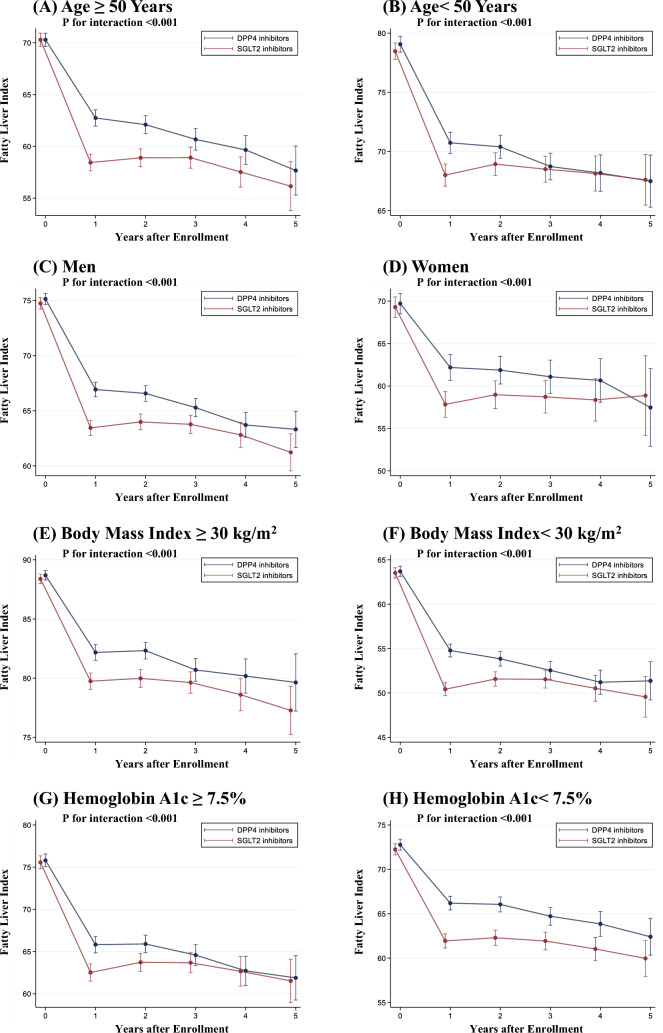
Subgroup Analysis. We performed a linear mixed-effects model to compare the change in fatty liver index among SGLT2 inhibitors and DPP4 inhibitors stratified by age, sex, body mass index, and hemoglobin A1c. The model included treatment group (SGLT2 inhibitors or DPP4 inhibitors), time, and the interaction between the treatment group and time. Error bars represented a 95% confidence interval

First, we analyzed 5783 SGLT2 inhibitor users and 5783 DPP4 inhibitor users who continued to use SGLT2 or DPP4 inhibitors for > 3 months. The main findings remained unchanged in this population (Supplementary Fig. 3). Second, we confirmed the robustness of the shape of the change in the FLI using the cubic spline function (Supplementary Fig. 4). Third, the results in terms of changes in FLI among SGLT2 and DPP4 inhibitors were generally consistent across subgroups stratified by age, sex, BMI, and HbA1c (Fig. 3).

## Discussion

In the present study, using a large-scale health checkup and claims database including approximately 20,000 patients with MAFLD and DM, we compared the change in FLI between SGLT2 and DPP4 inhibitors after propensity score matching. Administration of SGLT2 inhibitors was associated with improved FLI and liver enzymes (γ-GTP, AST, and ALT) and decreased BMI and waist circumference. Various sensitivity analyses have shown consistent results. These findings were observed across all subgroups stratified by age, sex, BMI, and HbA1c level at baseline. The association of SGLT2 inhibitors with amelioration of FLI was comparable between individual SGLT2 inhibitors, suggesting a potential class effect of SGLT2 inhibitors for DM and MAFLD. To the best of our knowledge, this is the first study to show the possible advantage of SGLT2 inhibitors used for MAFLD using a large-scale real-world dataset.

Several clinical trials have validated the effect of SGLT2 inhibitor administration on FLD. A prospective observational study included 21 patients with type 2 diabetes, and the use of ipragliflozin for 16 weeks was associated with a decrease in FLI, HbA1c, body weight, and visceral adipose tissue [24]. In the Liraglutide Effect and Action in Diabetes trial, a prospective, single-arm trial, 40 patients with type 2 diabetes and NAFLD were treated using luseogliflozin for 6 months. Treatment with luseogliflozin was associated with decreased HbA1c and transaminase activity, as well as improvements in hepatic fat content. Serum ferritin levels were reduced and serum albumin increased after treatment with luseogliflozin [25]. Further, a prospective randomized controlled pilot study included 32 patients with type 2 diabetes and NAFLD and randomly assigned study participants to receive either luseogliflozin or metformin. The changes in the liver-to-spleen attenuation ratio, changes in the visceral fat area, HbA1c, and BMI after 6 months were significantly greater in the luseogliflozin group than that in the metformin group [18]. The Effect of Empagliflozin on Liver Fat Content in Patients With Type 2 diabetes trial was an investigator-initiated, prospective, open-label, randomized clinical study and randomly assigned 50 patients to the empagliflozin (standard treatment for type 2 diabetes plus empagliflozin) or control groups (standard treatment for type 2 diabetes alone) for 5 months. The reduction in liver fat assessed using magnetic resonance imaging-derived proton density fat fraction was significantly greater in the empagliflozin group. Furthermore, the empagliflozin group showed a significant decrease in serum ALT levels [26]. A randomized, 48-week, open-label, active-controlled trial randomly assigned 40 patients with biopsy-confirmed NAFLD and type 2 diabetes to receive tofogliflozin or glimepiride. The fibrosis score was improved in the tofogliflozin-treated group. The histological variables of steatosis, hepatocellular ballooning, and lobular inflammation improved in the tofogliflozin group, whereas only hepatocellular ballooning improved in the glimepiride group [15].

Although the present study is in agreement with previous studies in that we demonstrated the potential benefit of SGLT2 inhibitors for FLD, our study is distinguishable from previous studies in the following points and has clinical implications. We analyzed approximately 20,000 patients with DM and MAFLD using a large-scale epidemiologic cohort and compared approximately 6,500 well-balanced pairs of new users of SGLT2 or DPP4 inhibitors with propensity score matching. Given that clinical trials investigating the effects of SGLT2 inhibitors on FLD have been limited to a maximum of approximately 50 patients, our study is the first to examine the association between SGLT2 inhibitor administration and outcomes using a large-scale real-world dataset. Several potential pathological mechanisms for the possible benefits of SGLT2 inhibitors for MAFLD have been suggested (e.g., calorie restriction, improvement in systemic insulin resistance, and reduction in body weight). In this study, there was a greater improvement in HbA1c in the SGLT2 inhibitor group than in the DPP4 inhibitor group. The improvement in glucose tolerance in the SGLT2 inhibitor group could contribute to the greater improvement in fatty liver as well. Currently, cardiovascular and kidney protective effects have been demonstrated for SGLT2 inhibitors in patients with and without DM, and the pathological or pharmacological mechanisms underlying these effects of SGLT2 inhibitors are attracting interest. Basic or experimental studies are needed to determine the hepatoprotective effects of SGLT2 inhibitors. Furthermore, we need to clarify whether SGLT2 inhibitors could provide clinical benefits for patients with MAFLD without DM. On the other hand, the differences between the SGLT2 and DPP4 inhibitor groups were seemingly attenuated over time. Changes in medication (e.g., addition of SGLT2 inhibitors to the DPP4 inhibitor group) and lifestyle modifications during the follow-up period could have contributed to these results. Further data accumulation and investigation are needed in this regard. The large sample size of our database allows for various sensitivity analyses to confirm the robustness of our findings. In particular, it is important that the various subgroup analyses stratified by age, sex, BMI, and HbA1c level suggest a potential advantage of SGLT2 inhibitors over DPP4 inhibitors. We found that the influence of SGLT2 inhibitors on MAFLD would be similar among individual SGLT2 inhibitors, suggesting a potential “class effect” of SGLT2 inhibitors, which is consistent with our previous studies [21, 27]. As it may not be feasible to conduct a randomized controlled trial in this point of view, we believe that our study could have provided intriguing clinical data.

We acknowledge the study limitations mainly due to the use of the JMDC Claims Database, which we previously discussed [21, 27]. Because of the observational and retrospective nature of the present study, and despite robust statistical procedures, including propensity score matching and a multitude of sensitivity analyses, the possibility of unmeasured residual confounding could not be eliminated. For instance, although socioeconomic status or the duration of DM could have affected clinical outcomes, the JMDC Claims Database did not include these data. Because most of the people registered in our dataset are employees (or their family members) who work for relatively large companies in Japan, the socioeconomic status of the study participants would not be significantly different. However, the absence of these data must be considered a potential limitation of our study. It is unknown whether our findings may be applied to the older population because the JMDC Claims Database does not contain those over 75 years of age. We did not take into account the dosage of each medication. We evaluated the effects of SGLT2 inhibitors on liver enzyme levels, BMI, and waist circumference. However, it should be considered that liver enzymes alone may not reflect the liver histological responses. There is a possibility of treatment changes during the clinical course following the initiation of SGLT2 or DPP4 inhibitors, and this should be considered as a potential factor that could have influenced the results of the present study.

## Conclusion

In real-world clinical practice, patients with MAFLD and DM who were newly prescribed SGLT2 inhibitors showed significantly better improvement in liver enzymes, reduction in BMI and waist circumference, and a decrease in FLI than those who were newly prescribed DPP4 inhibitors. Our findings were consistent irrespective of age, sex, BMI, and HbA1c level at baseline. This study, using a large-scale epidemiological cohort, complements the results of previous randomized clinical trials and confirms the potential liver-protective benefits of SGLT2 inhibitors in patients with MAFLD and DM.

### Supplementary Information

Below is the link to the electronic supplementary material.

Supplementary file1 (PPTX 193 KB)

## Data Availability

The JMDC Claims Database is available for purchase from JMDC Inc. (https://www.jmdc.co.jp/en/).
