# Supplementary material for: Comparison of SGLT2 inhibitors vs. DPP4 inhibitors for patients with metabolic dysfunction associated fatty liver disease and diabetes mellitus
Source: J Endocrinol Invest. 2023 Dec 19;47(5):1261–70. doi: 10.1007/s40618-023-02246-6 (PMC11035461; doi:10.1007/s40618-023-02246-6)
Supplement: Supplementary file 1 — Supplementary file1 (PPTX 193 KB) [file 40618_2023_2246_MOESM1_ESM.pptx]

## Slide 1
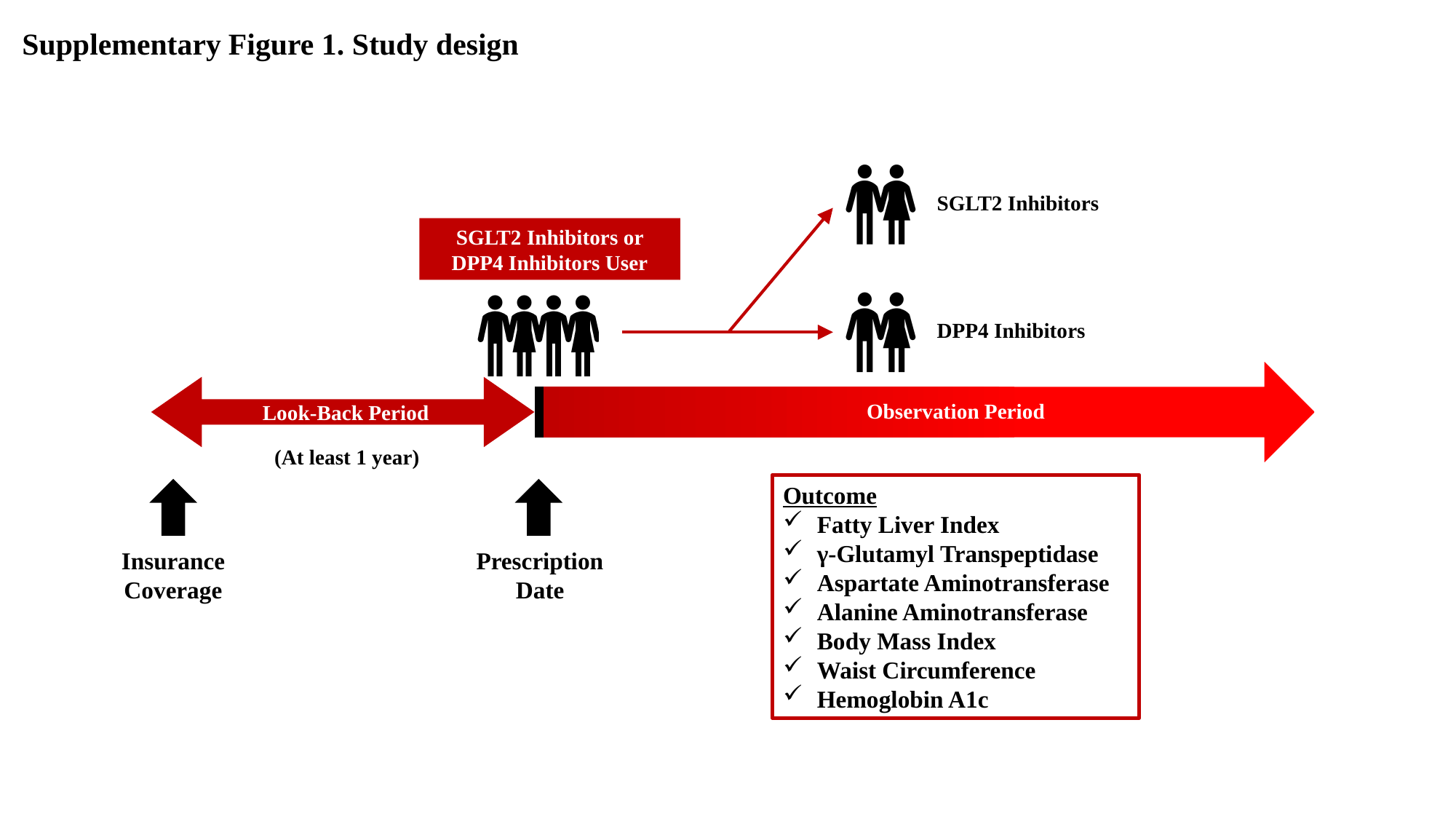

Supplementary Figure 1. Study design
SGLT2 Inhibitors
SGLT2 Inhibitors or DPP4 Inhibitors User
DPP4 Inhibitors
Observation Period
Look-Back Period
(At least 1 year)
Outcome
Fatty Liver Index
γ-Glutamyl Transpeptidase
Aspartate Aminotransferase
Alanine Aminotransferase
Body Mass Index
Waist Circumference
Hemoglobin A1c
Prescription
Date
Insurance
Coverage

## Slide 2
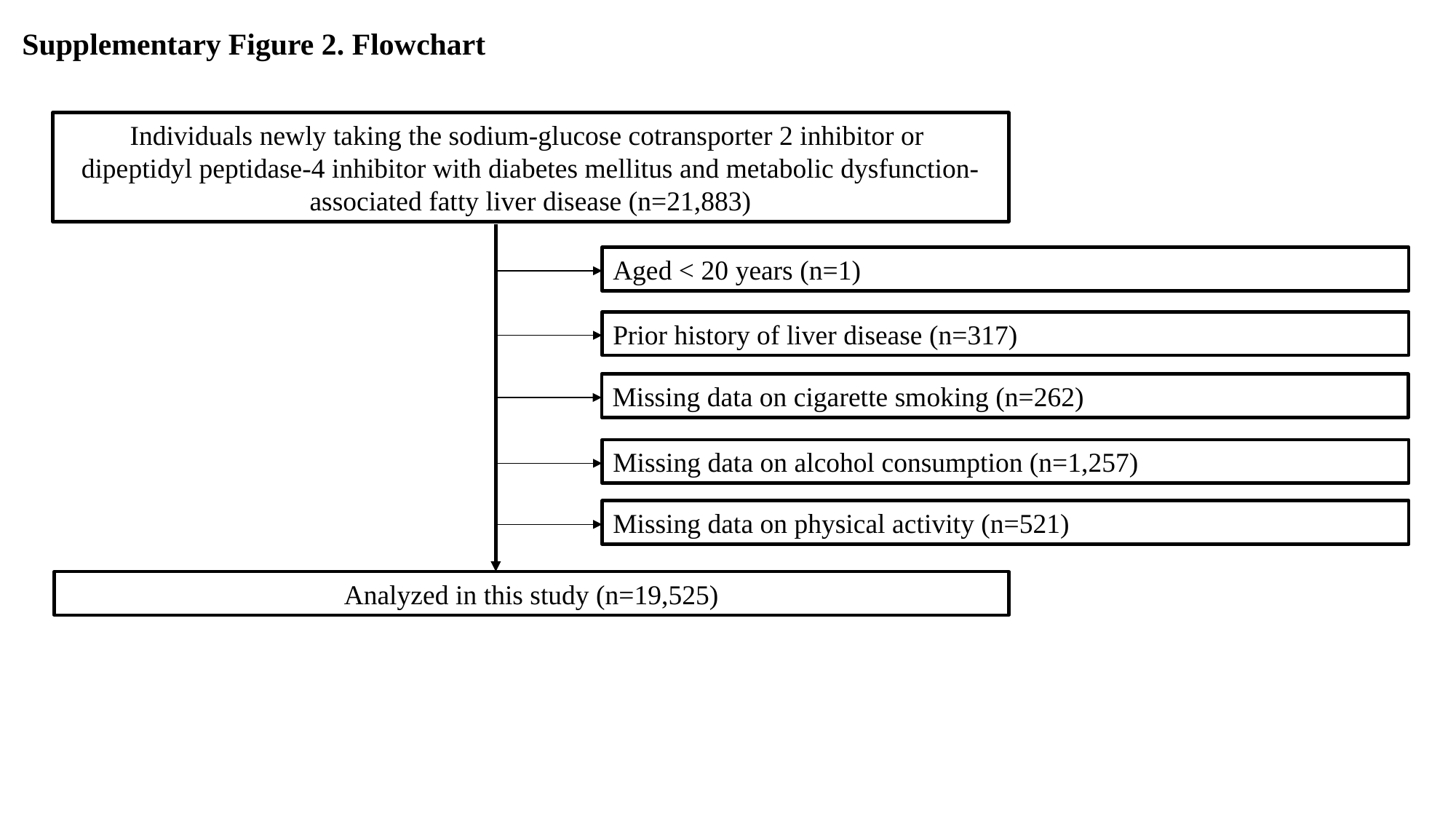

Supplementary Figure 2. Flowchart
Individuals newly taking the sodium-glucose cotransporter 2 inhibitor or
dipeptidyl peptidase-4 inhibitor with diabetes mellitus and metabolic dysfunction-associated fatty liver disease (n=21,883)
Aged < 20 years (n=1)
Prior history of liver disease (n=317)
Missing data on cigarette smoking (n=262)
Missing data on alcohol consumption (n=1,257)
Missing data on physical activity (n=521)
Analyzed in this study (n=19,525)

## Slide 3
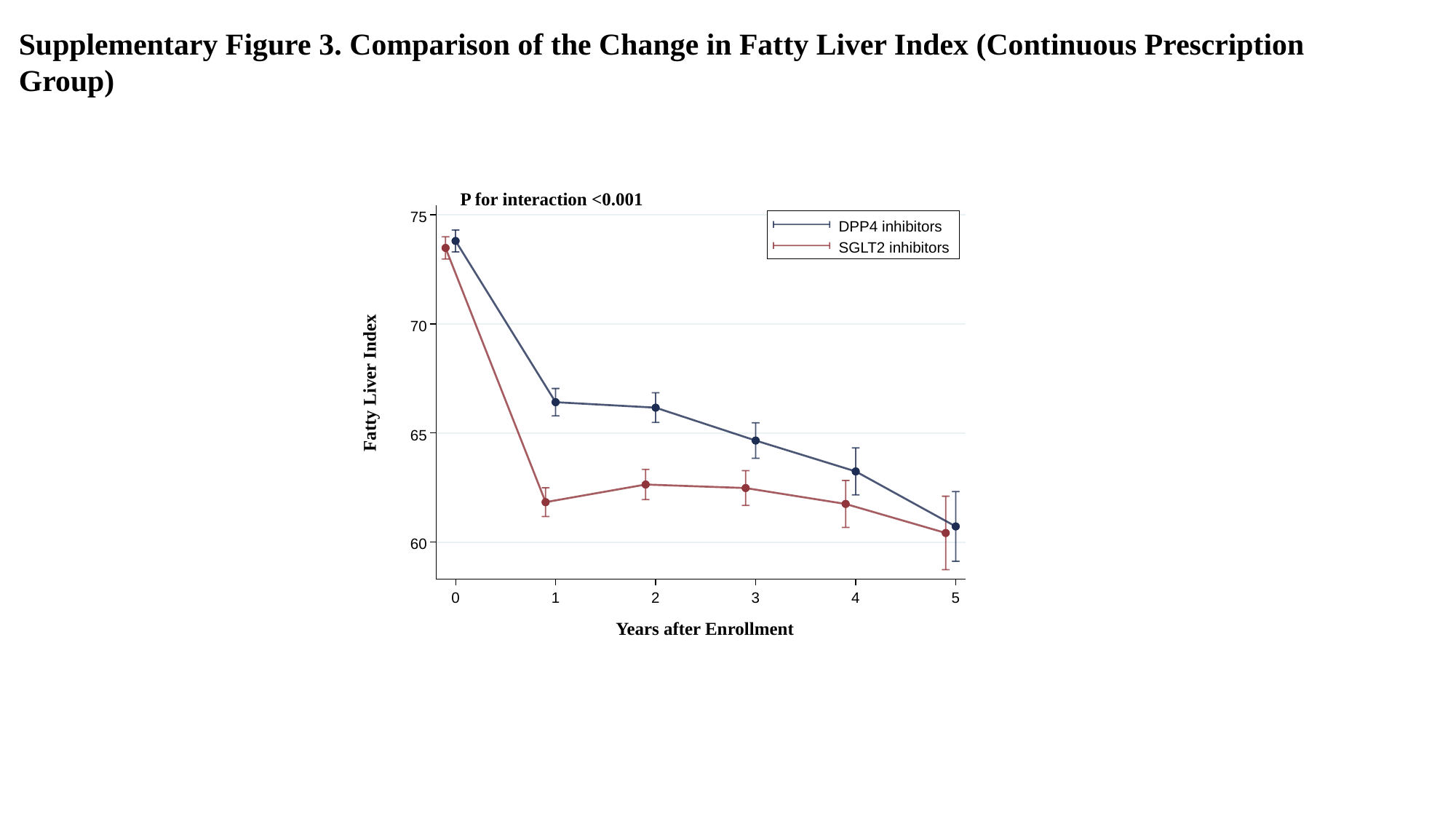

Supplementary Figure 3. Comparison of the Change in Fatty Liver Index (Continuous Prescription Group)
P for interaction <0.001
Fatty Liver Index
Years after Enrollment

## Slide 4
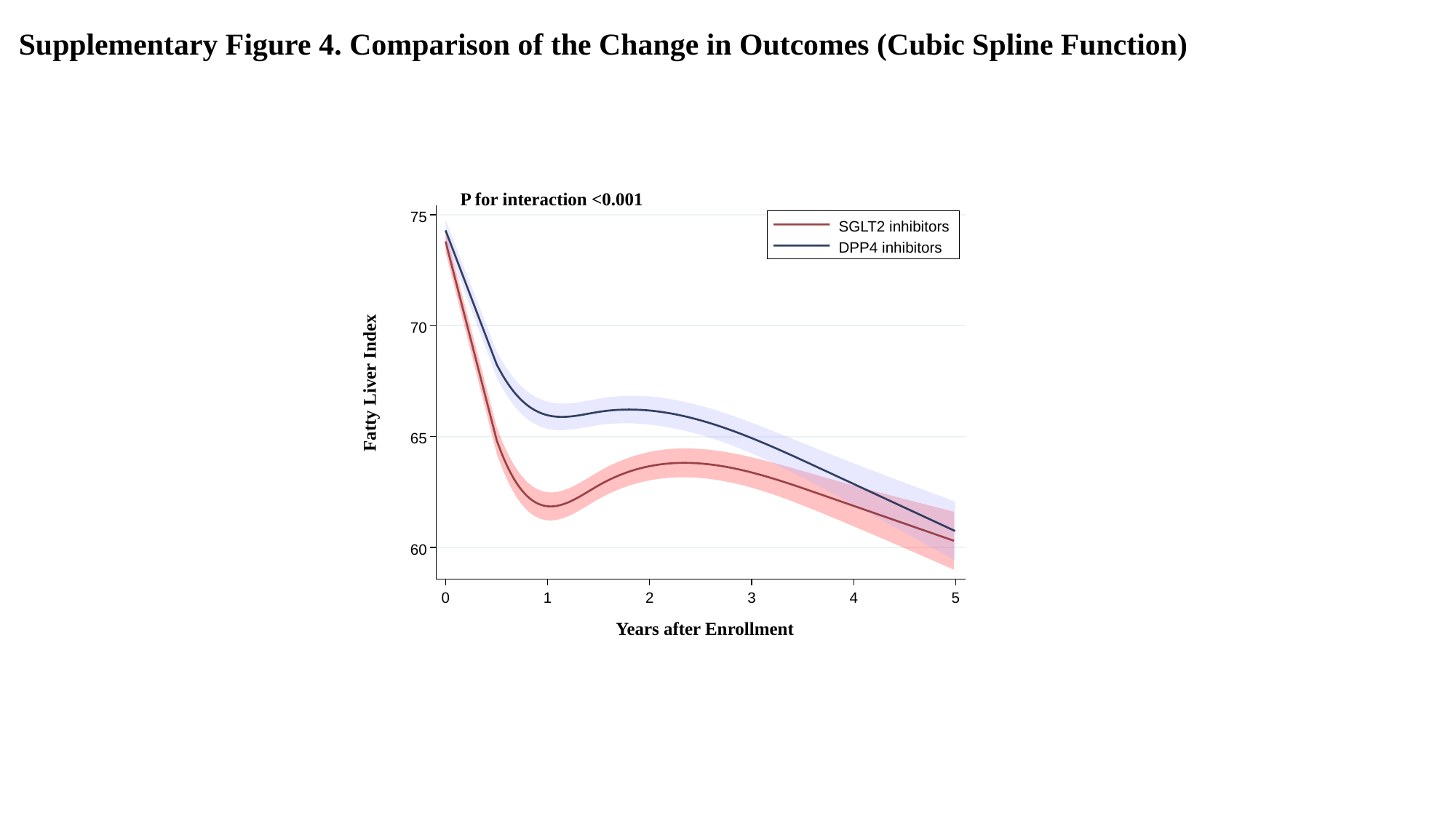

Supplementary Figure 4. Comparison of the Change in Outcomes (Cubic Spline Function)
P for interaction <0.001
Fatty Liver Index
Years after Enrollment
